# Thermodynamic Data Remain a Hot Tip for Decoding Binding Affinity and Water Impact on Protein–Ligand Complex Formation to Assist Lead Optimization

**DOI:** 10.1021/acs.jmedchem.5c03100

**Published:** 2026-02-17

**Authors:** Gerhard Klebe

**Affiliations:** Institute of Pharmaceutical Chemistry, 9377 Philipps University Marburg, Marbacher Weg 6, Marburg 35032, Germany

## Abstract

Optimization of screening hits from lead-finding campaigns into promising lead candidates can be supported by factorizing thermodynamic binding profiles into enthalpic and entropic contributions. Given that data are recorded for large multicomponent systems, typically affected by substantial enthalpy/entropy compensation, it is exceedingly challenging to directly assign enthalpy and entropy to rational design concepts. Correcting recorded data for superimposed protonation steps and analyzing structural and dynamic properties is essential. Subsequently, thermodynamic signatures can be assigned to related ligand pairs. However, profile-determining contributions can vary from case-to-case, even steps prior to protein binding can be determinant. Pocket solvation, prior to or during ligand binding, can have substantial influence on binding profiles, leading to more enthalpy or entropy-driven profiles. Since a range from dry to well-solvated pockets is observed, different thermodynamic signatures can be recorded. The quality of newly formed surface-solvation shells generated after ligand binding can significantly impact ligand affinity.

## Graphical abstract

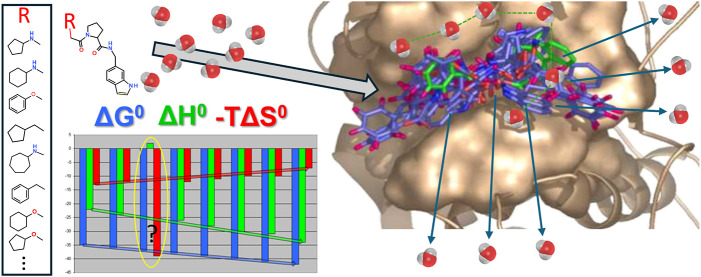

## Introduction

In a drug discovery project, hits from a lead-finding campaign are typically optimized into promising lead candidates using rational design concepts. These concepts then drive subsequent medicinal chemistry syntheses. Lead optimization campaigns must consider multiple parameters simultaneously in order to transform a pharmacologically interesting organic molecule into an administrable drug. These parameters include sufficient potency toward the biological target, balanced solubility, permeability, metabolic stability, selectivity, tolerable toxicity, and functional efficacy. In the early stages of a project, potency or affinity is usually considered a key property for tracing and validating the success of subsequent design steps. This contribution will focus on ligand affinity toward its target. However, it must be kept in mind that in a drug development project, the highest achievable affinity does not necessarily translate into sufficient *in vivo* efficacy. Therefore, affinity must be considered within the broader context of attempted multiparameter optimization.

However, what kind of a quantity is ligand affinity? It is usually reported as a binding constant (*K*_i_, *K*_d,_ or *K*_a_) or at least as an *IC*_50_ value within a congeneric compound series.^1,2^ But what are the rational considerations that drive affinity enhancement? Defined as an equilibrium quantity, affinity is expressed as a binding constant and is logarithmically related to the Gibbs free energy of binding ΔG^0^ (Δ*G* ^0^ = −RT ln *K*_d_). The latter quantity decomposes additively into an enthalpic Δ*H* ^0^ and entropic contribution −*T*Δ*S* ^0^, where the entropic part is weighted by the applied temperature on an absolute scale.

This immediately raises the question whether knowledge of this decomposition can support the design strategy to be selected.^3–5^ Methods to record thermodynamic data are available, in particular isothermal titration calorimetry (ITC).^6,7^ This method is performed in solution and does not need any labeling. It has furthermore the advantage that information about Δ*H* ^0^ and −*T*Δ*S* ^0^ becomes available in one experiment at a given temperature (strictly speaking *K*_d_ and Δ*H* ^0^, the entropic part is taken from the difference). Nevertheless, we have to consider that the studied system is composed of multiple components. In the simplest case we have to consider the ligand, the protein and a large number of water molecules. These are located in the bulk water phase, locally surrounding the binding partners, or they are bound at their surfaces and occupy pockets which are accessible from the surface by some protein-specific binding mechanisms. Is there any realistic chance to learn something meaningful about such a complex multicomponent system by simply recording two overall thermodynamic properties that capture all changes involved in the binding process? Is this not an *a priori* impossible undertaking, doomed to failure? It is therefore extremely important to keep the experiments as simple as possible, and, in any case, to make measurements at only one temperature (i.e., no van’t Hoff evaluations across a set of different temperatures which unavoidably will further complicate the system caused by numerous overlaid temperature-dependent effects).^1,2^

This option is provided by the ITC method. But is this enough? In our experience, the analysis remains very difficult. Therefore, as much information as possible about the studied system needs to be collected. Furthermore, it is necessary to check for additional overlaid processes, such as the exchange of protons between the binding partners and the surrounding buffered medium during the binding process.^8–11^ This can be done by recording the binding from buffers with different ionic strengths. Ligands **1** and **2** in Table 1 were measured from different buffer solutions. Whereas **1** picks up a proton upon binding, **2** remains uncharged and no buffer dependence is observed. For ligand **1** it matters a lot which buffer is used for the measurement. Therefore, corrections are needed: at the very least, it must be checked whether the same protonation step occurs for all ligands investigated in an optimization series, e.g., on the protein side. Then, in a relative comparison the protonation effect on the side of the protein cancels out because the same amount occurs for the binding of all ligands in a series. In addition, a strict control of the adopted ligand binding poses is absolutely necessary using a structural biology method such as crystallography. How many structural changes occur during binding, even for ligands that appear structurally similar at first glance? Molecular dynamics (MD) simulations are often required to verify changes in individual structural parameters during binding and to trace possible changes due to the dynamic properties of the studied system. Do any of the ligands exhibit exceptional conformational properties in solution before binding to the protein? Since a specific thermodynamic profile is to be correlated with the structural change in an applied optimization step, it is important to ensure that only this step is primarily responsible for the change from one ligand to the next and expresses itself in the thermodynamic signature. A relative comparison between two ligands, i.e., by considering ΔΔG^0^, ΔΔ*H* ^0^ and −*T*ΔΔ*S* ^0^, can then be used to interpret the results.

**Table 1:**
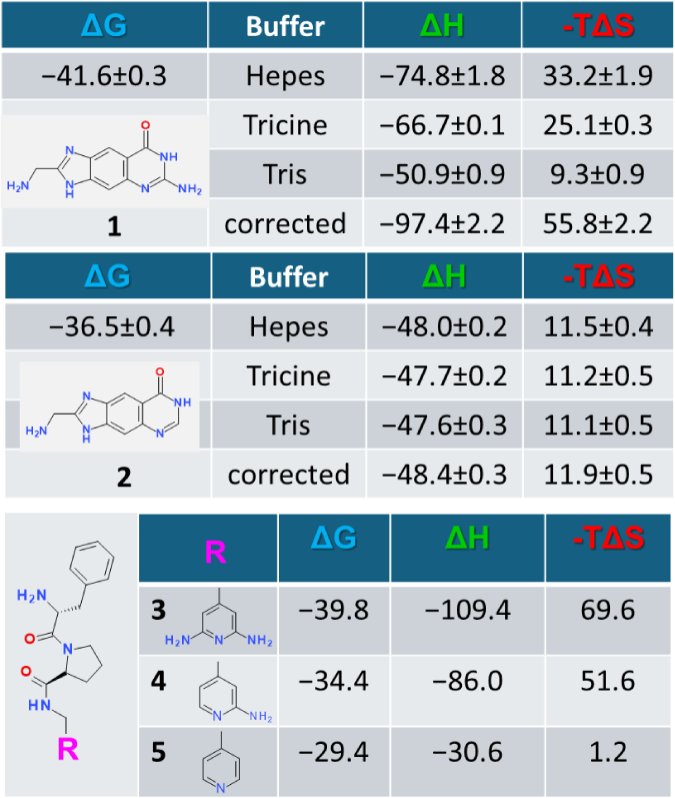
Thermodynamic Data of Five Ligands Binding to Either tRNA Guanine Transglycosylase (**1**, **2**) or Thrombin (**3**–**5**)

These concerns may lead one to conclude that thermodynamic analysis is not feasible for routine practical use, especially in industry, because it is too complex, too time-consuming and requires far too much additional background information about the system. Nevertheless, to our experience, once some series of ligands has been evaluated in this way, general principles emerge that can be used as working hypotheses in the design of future optimization projects. Furthermore, if one member of a congeneric ligand series shows a strongly deviating profile from the others, the likelihood of differences in its binding profile are obvious and needs to be checked. If these aspects are given proper consideration, the approach certainly has considerable potential. This perspective aims to examine and summarize a number of insights resulting from this concept.

## Data Source, Data Quality, and Correction

As previously mentioned, a prerequisite for the desired analysis, which seeks the parameters that determine the thermodynamic profile of ligand binding, is collecting and correcting multiple data points of protein–ligand complexes in a highly controlled and uniform manner. To meet this criterion, it is necessary to have full access to and control over the data to be evaluated. Therefore, for this perspective, the author used data collected in his research group over the past 20 years because the data have been consistently collected and validated, and the same criteria have been applied throughout. For this reason, the analysis will focus on these data only. This should not be seen as a disregard for the work of others, and these achievements are well acknowledged in the comprehensive annual reports published in *J. Mol. Recognition*.^12–23^ The required ITC measurements, corrections, and crystal structure determinations, as well as computer simulations, were performed on the following seven enzymes: aldose reductase, human carbonic anhydrase II, protein kinase A, thermolysin, thrombin, trypsin, tRNA guanine transglycosylase (see Table S1 along with the corresponding literature references). Many of the studied ligand series were synthesized for the purpose.

Admittedly, restricting the analysis to enzymes means that only a part of the current target landscape is considered. However, enzymes are the easiest and most accessible systems with respect to complexity. They are catalysts that bind tightest the transition state of a chemical reaction. This makes them ideal candidates for studying by equilibrium thermodynamics. Contemporary targets, such as GPCRs, ion channels, RNA, and protein–protein interaction interfaces, including PROTAC and molecular glue strategies, are often highly dynamic and sometimes poorly ligandable. Thermodynamic data on these targets are scarce, and since their pharmacological properties are largely governed by kinetic and dynamic aspects, such as the opening frequency and duration of an ion channel, or the dynamic shifting of conformational equilibria between agonistic and antagonistic behavior, these targets represent unconventional modalities. The question remains as to how much can be extracted from equilibrium thermodynamics on such complex systems. Therefore, here, we will focus on enzymes as targets.

The current evaluation includes a total of 266 data points. Ligand data were validated and if necessary corrected for superimposed changes in protonation steps. As mentioned above, ligand **1** picks up a proton upon protein binding whereas the structurally related **2** remains uncharged (Table 1). In consequence for **1** the partitioning into enthalpic and entropic binding contributions strongly depends on the buffer system chosen for the measurement, whereas the data of **2** are buffer independent. A change in protonation state of either the protein, ligand, or buffer, involves a “heat-of-ionization” of the functional groups releasing or picking up the proton, which is overlaid on the measured heat signal of the binding process. Thereby the heat-of-ionization effects are minor for oxygen-containing functional groups, whereas larger values are found for nitrogen-containing functional groups. Therefore, phosphate and acetate buffers are ideal to minimize buffer-dependent heat effects. When a protein residue takes up or releases a proton without directly interacting with the bound ligand, the data from one buffer are sufficient for analyzing the relative differences in the ligands’ binding signatures because the impact on enthalpy and entropy is the same for all ligands in a series. For instance, ligand binding to thrombin is consistently accompanied by His 57 taking up half a mole of protons in the binding reaction. However, this effect may be enhanced or nullified if a ligand is chosen that also picks up or releases a proton. Such effects can be surprising, even in congeneric series of ligands.^24^ Furthermore, a set of 55 fragments was uniformly studied in acetate buffer via displacement titration using for all complexes the same displacement ligand.^25^ Here, endothiapepsin was chosen as target protein, because large quantities of this protein are readily available on a gram scale. The weak ITC heat signal associated with fragment binding can only be analyzed by using very large amounts of target protein.^26,27^

### Data Representation and Analysis

The data of the 266 ligand-protein complexes (blue circles) and 55 endothiapepsin fragment complexes (magenta circles) are displayed in a Δ*H* ^0^ versus −*T*Δ*S* ^0^ diagram (Figure 1a, left). The diagram is divided into a red and green area, which should indicate the regions where either enthalpy (green) or entropy (red) dominates binding affinity.^2,28^ Along the borderline between the red and green regions, the enthalpic and entropic contributions are equal. Increasing ΔG^0^ contributions of the individual complexes are parallel to the main diagonal and variations in ΔG^0^ are limited to a range of about −10 to −55 kJ/mol. This reflects the range available to medicinal chemists to optimize their complexes. Perpendicularly, the data for the complexes scatter over a more than 6 times larger range of Δ*H* ^0^ (here: −110 to 0 kJ/mol) and of −*T*Δ*S* ^0^ (here: −40 to +70 kJ/mol) without achieving an improvement in ΔG^0^. This large scatter indicates the well-known enthalpy/entropy compensation phenomenon.^29–33^ Although there is no physical relationship that defines the mutual compensation of these two thermodynamic properties in protein–ligand complexes, this observation shows that optimizing one property is often offset by a compensating effect of the other property.^34–36^ This underscores the difficulty of optimizing a given protein–ligand complex, as the compensating effects remain undiscovered as long as only information about affinity (or ΔG^0^) is measured. Therefore, predicting how molecular changes affect affinity and the resulting thermodynamic signature requires information on Δ*H* ^0^ and −*T*Δ*S* ^0^ along with additional background information such as structural data and molecular dynamics simulations for a sophisticated planning of the next optimization steps.^34,37–39^ As mentioned above, first of all correcting superimposed protonation steps is important.^8–11^ Buffer-uncorrected data from complexes where titratable groups can change their protonation state contributes to enthalpy/entropy compensation, as illustrated by the examples **1** and **2** in Table 1. Corrected data emphasize that **1** is a strong enthalpic binder. However, based on data collected in Tris buffer, the enthalpic signature of **1** would appear much smaller. Interestingly, the data for the fragment complexes (magenta) with endothiapepsin fall within a narrow window of about 10–20 kJ/mol for ΔG^0^, but the scatter of the data for Δ*H* ^0^ and *–T*Δ*S* ^0^ is similar to that of larger, more potent protein-drug-like ligand complexes, at least for the proteins considered in this study. Figure S1 shows color-coded data for the individual proteins. The largest data set has been collected for thrombin, and data scatter is found across the entire range. The same is true for the enzyme tRNA guanine transglycosylase. Aldose reductase data spread across the entire enthalpic range, whereas thermolysin and carbonic anhydrase complexes cluster within a smaller, mostly enthalpic area. This clearly reflects the chemistry of the analyzed ligands and enzymes. For example, in the case of carbonic anhydrase, it should be noted that binding to the zinc ion accounts for approximately half of the affinity for complex formation. Since all of the considered complexes use a sulfonamide anchor, the scatter of the data is small for the studied complexes (Figure S1).

**Figure 1:**
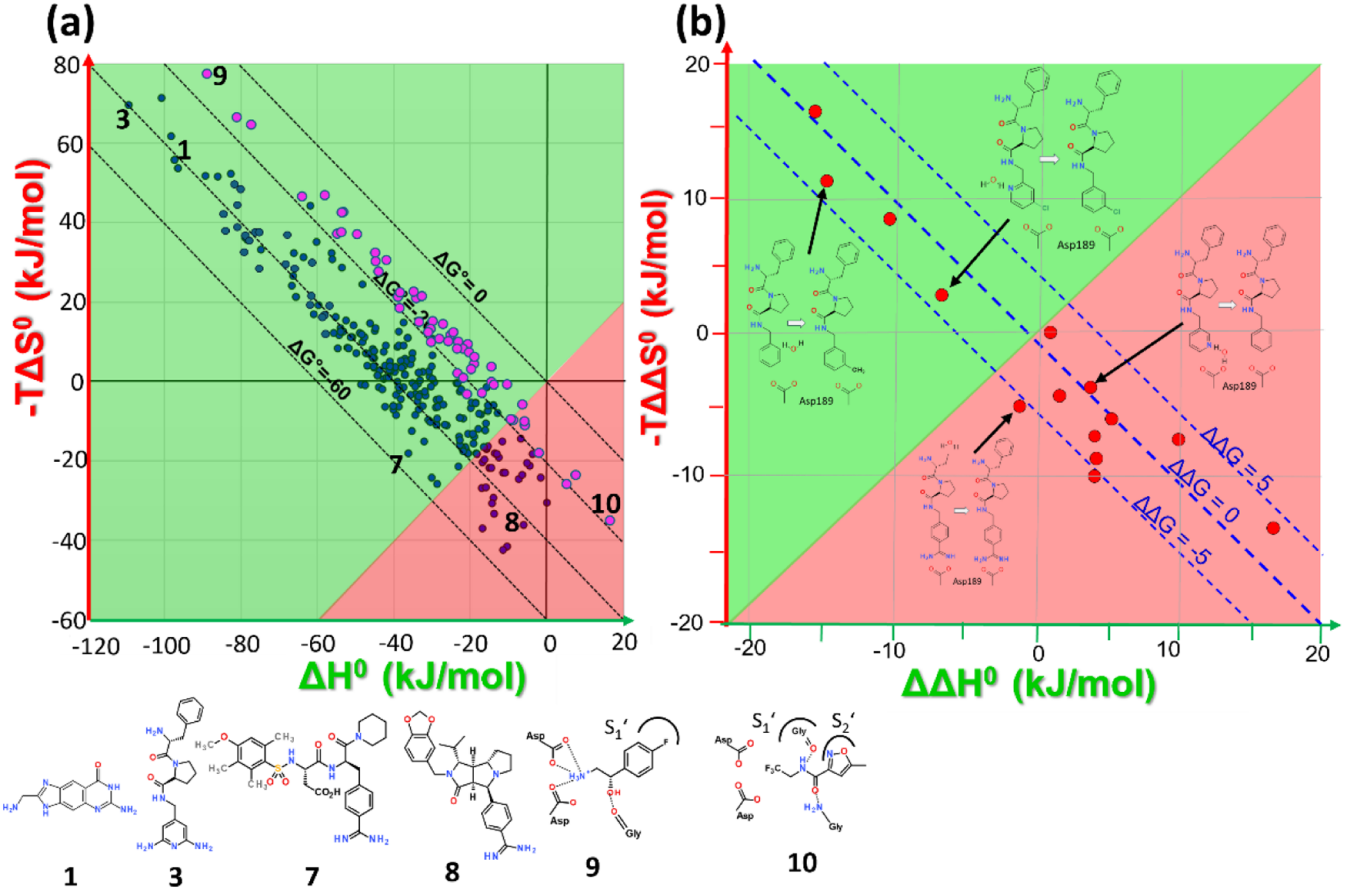
(a, left) The ITC data of 266 protein–ligand complexes (blue spheres) and 55 endothiapepsin-fragment complexes (magenta spheres) are plotted in a Δ*H* ^0^ versus *–T*Δ*S* ^0^ diagram. The data were collected using seven different enzymes: thrombin, trypsin, thermolysin, carbonic anhydrase II, aldose reductase, tRNA guanine transglycosylase, protein kinase A, and fragment data exclusively collected with endothiapepsin. The diagram is divided into a green and red area, where either the enthalpic (green) or entropic (red) component dominates binding. Along the main diagonal, the Gibbs free energy of binding (ΔG^0^) improves from approximately −10 kJ/mol to −55 kJ/mol, which is the range usually covered by medicinal chemistry optimizations. The data perpendicular to the main diagonal scatters over a range more than six times larger, where enthalpy and entropy show opposing contributions to ΔG^0^, resulting in a mutual compensation of both properties (so-called enthalpy/entropy compensation). The ligand binding data were checked and corrected for superimposed protonation effects during binding. For all complexes, the binding pose was validated by crystal structure analysis. The chemical formulas of some ligands in the complexes are indicated and can be traced in the diagram by the assigned numbers. (b, right) For 14 pairs of complexes (Table S2) shown on the right where binding mainly differs by the pick-up or release of a water molecule in the binding site are depicted as relative differences across the pairs in a ΔΔ*H* ^0^ versus *–T*ΔΔ*S* ^0^ diagram. As on the left, the diagram has been split into a red and green area indicating predominantly enthalpic or entropic binding. For the shown examples modulation of the Gibbs free energy ΔΔG^0^ scatters over a small range, however as for the complexes a huge compensation of enthalpic and entropic binding contributions are observed. The chemical formulas for some selected pairs of ligands are depicted.

It is interesting to see which complexes correspond to the extremes in the diagram. Complexes with a dominant enthalpic binding signatures have polar and likely charged groups enhancing hydrogen bonding in deeply buried protein pockets (Figure 1a, **1** or **3**). The most entropically binding complexes are formed with rigid ligands comprising a reduced number of rotatable bonds and being decorated with hydrophobic groups (e.g., **8**). In the fragment data set, the most enthalpic binder positions a charged group next to the two catalytic aspartate residues (**9**). In contrast, the most entropic binder accommodates the specificity pockets S_1_′ and S_2_′ of endothiapepsin and forms only one hydrogen bond from its peptide NH group to a glycine residue’s carbonyl oxygen (**10**). These observations suggest that state-of-the-art medicinal chemistry optimizations, such as increasing molecular size, performing correct rigidifications, or attaching more hydrophobic, mostly aromatic substituents, will result in an increasing entropic binding. However, these optimizations are often linked to a reduced resistance profile, decreasing water solubility and increasing unspecific toxicity.^34–36,40^ These aspects are important to prevent. Since enthalpy/entropy compensation is difficult to avoid, would it not be better to start with a more enthalpic binder to prevent early deterioration due these detrimental properties? ^34,40^ However, as will be shown, this seemingly attractive and intuitive strategy requires careful considerations because the features determining the finally measured overall binding signature are more complicated and involve many more factors than the ones listed above.

### Increasing Enthalpic and Entropic Signatures to Improve Affinity

To trace the details of changes in the binding profile, particularly to study whether binding is more enthalpy or entropy-driven, we will consider the relative changes in the thermodynamic profile (so-called ΔΔ*X* ^0^ values, *X* = *G*, *H*, or *S*) between closely related pairs of complexes where crystallographic analysis has confirmed unchanged binding modes and that possibly overlaid effects resulting from changes in protonation states have been considered.

The first example involves occupancy of the deeply buried S_1_ pocket in thrombin, which contains a charged aspartate residue at its far end. Ligand **3** (Table 1) forms a strong charge-assisted hydrogen bonding network with the aspartate 189 through its diortho-diamino pyridine moiety and shows the strongest enthalpic signature of all complexes in Figure 1 (Table 1).^41^ A significant enthalpic enhancement is observed compared to the unsubstituted pyridine derivative **5**, which even binds in an uncharged state. The monoamino derivative **4** is less potent, yet it still experiences a strong enthalpic signal comparable to **3**. In the enzyme tRNA guanine transglycosylase, the aminopyrimidone moiety of **1** binds deeply in a polar pocket in charged state (Table 1). The analogous hypoxanthine derivative **2** binds with lower potency but similar binding geometry. It remains uncharged and exhibits a much smaller enthalpic signature.^42^

The introduction of a five-membered ring restricts conformationally the thrombin inhibitor **11** (Figure 2, *upper row, far left*). Consequently, compound **12**, which is now correctly rigidified for the bound state, exhibits higher potency resulting from a strongly enhanced entropic signature (−*T*ΔΔ*S* ^0^_**10⇒11**_ = −7.8 kJ/mol).^43^ A similar enhancement is observed in the series **13**-**15** (Figure 2, *upper row, center left*).^44^ All three ligands adopt a very similar overall geometry in the protein binding pocket of thrombin. The open-chain 4-amidinobenzyl derivative **13** is stabilized in **14** by intramolecular hydrogen bonds formed between the NH_2_ group of the 2-(aminomethyl)-5-chlorobenzyl substituent and two side-chain carbonyl oxygen atoms. Interestingly, the analogous OH derivative **15** does not exhibit this affinity enhancement and entropic advantage (−*T*ΔΔ*S* ^0^_**13⇒14**_ = −7.2 kJ/mol; −*T*ΔΔ*S*_**13⇒15**_ = −0.9 kJ/mol), despite adopting the same geometry as **14** at the protein binding site. MD simulations in solution show that **14** preorganizes into the bound conformation already prior to protein binding, whereas **15** is conformationally flexible and lacks favorable preorganization since the hydrogen bonds to the OH group in **15** are less stable than those to the NH_2_ group in **14** in aqueous solution. This difference prior to protein binding determines the observed overall thermodynamic signature.

**Figure 2:**
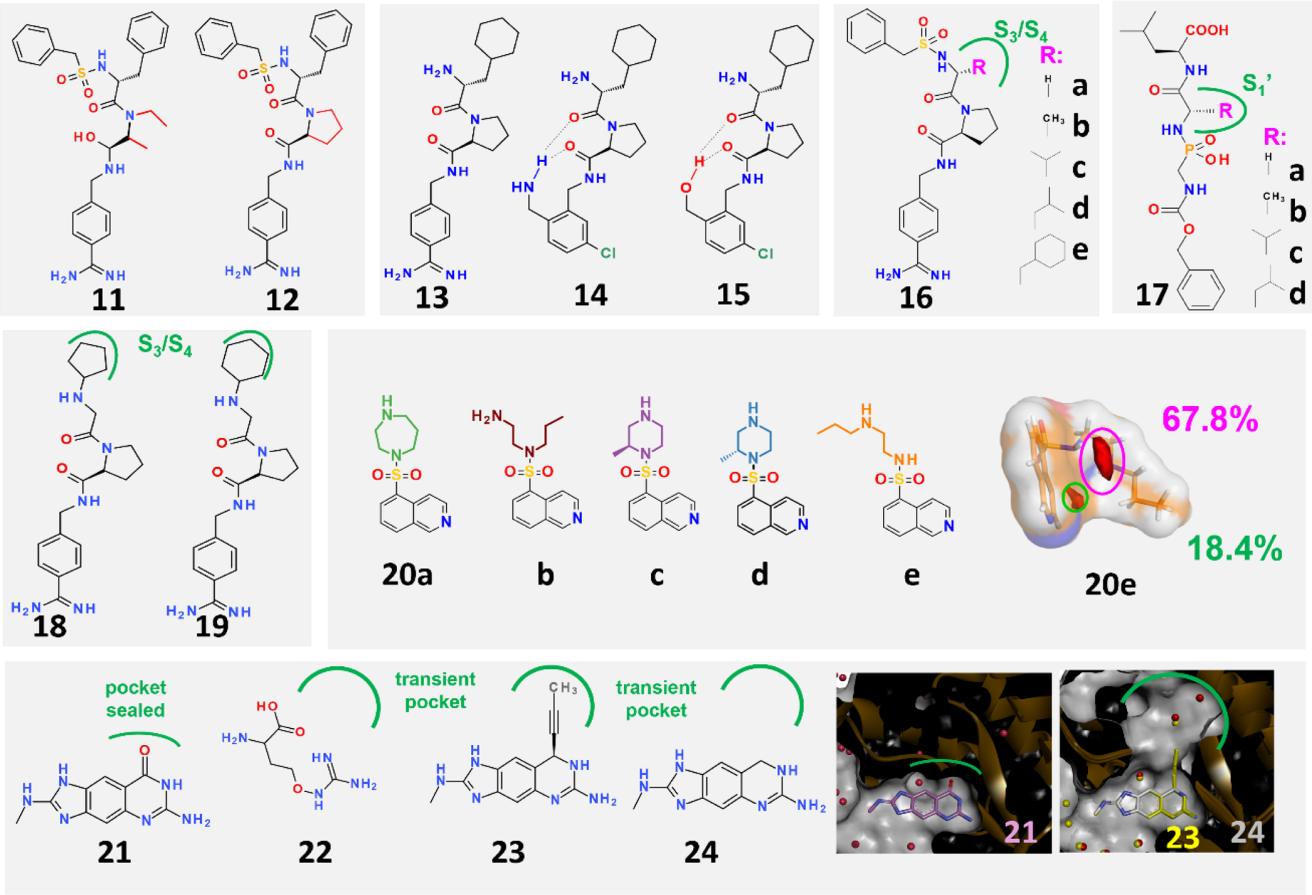
Chemical formulas of ligand pairs are used to relate enthalpic and entropic binding contributions to ΔΔG^0^. *Upper row, far left*: Ligand **12** has an entropic advantage over **11** when bound to thrombin because **12** is correctly fixed in the bound conformation in solution prior to protein binding. *Upper row, center left:* Compared to open-chain ligand **13**, **14** and **15** bind to thrombin with an intramolecular hydrogen bond. However, only **14** shows an entropic advantage over **13** because it adopts the preorganized conformation required for the bound state already in solution. The hydrogen bond of **15** and thus the bound conformation is not stable in solution. *Upper row, center right:* Ligand scaffold **16** was decorated with hydrophobic substituents (**a**–**e**) of increasing size to occupy the S_3_/S_4_ pocket of thrombin. These substituents demonstrate an increasing entropic binding profile due to the successive displacement of ordered water molecules. *Upper row, far right:* A series of thermolysin inhibitors **17a**-**e** which increasingly fill the S_1_′-pocket of the enzyme. From **17a** to **17e** an huge enthalpy-dominated affinity enhancement is observed. *Middle row, left*: Ligands **18** and **19** differ only in their terminal five- or six-membered cycloaliphatic substituents. The cyclopentyl moiety is well-defined in the electron density of the crystal structure, but the cyclohexyl ring is completely disordered. Different thermodynamic signatures result from the distinct residual mobility of the two substituents in the bound state. *Middle row, right*: Interestingly, ligand **20e**, which has the largest number of rotatable bonds, exhibits the most favorable entropic binding profile of **20a** to **20e**. Ligand **20e** captures and binds tightly to a water molecule in a bent, back-folded conformation. The water-binding sites are encircled in magenta and green, and their population percentages are shown. The release of the water molecule is critical to the binding signature. *Bottom row*: Usually ligands such as **21** bind to the catalytic center of tRNA guanine transglycosylase with the subpocket in closed state. Fragment **22** opens a transient pocket that becomes flooded with water molecules. Ligand **23** binds to this pocket, with its propyn-1-yl substituent replacing some of the water molecules. However, it does not have an increased binding affinity compared to **24**. On the right the ligands **21** (magenta, closed pocket) and **23**, **24** (yellow and gray, open pocket) are shown in atom-type color-coded stick model, the protein is shown with a white solvent-accessible surface (inside black), and its backbone is depicted as a dark-brown ribbon model.

In a series with a 4-amidinobenzyl anchor (**16**) for the S_1_ pocket (Figure 2, *upper row, center right*), the hydrophobic substituent occupying the hydrophobic S_3_/S_4_ pocket of thrombin has increased in size from a hydrogen (**a**) to a methyl (**b**), isopropyl (**c**), and *sec*-butyl (**d**) to a cyclohexylmethyl (**e**) substituent.^45^ This leads to the subsequent displacement of two crystallographic water molecules from the S_3_/S_4_ pocket accompanied by an increased hydrophobicity of the substituent and an enhanced entropic signature (ΔΔ*H* ^0^_**16a⇒16e**_ = 2.9 kJ/mol; −*T*ΔΔ*S* ^0^_**16a⇒16e**_ = −11.7 kJ/mol). This observation is usually explained by the classical hydrophobic effect.^46–49^ However, hydrophobic binding with an enthalpic signature can also occur.^50–53^ In an inhibitor series (**17**) for thermolysin (Figure 2 *upper row, far right* and Figure S2), the S_1_′ pocket substituent was optimized from hydrogen (**a**) to *sec*-butyl (**d**).^54^ The binding affinity increases by a factor of 41,000, primarily due to an increase in binding enthalpy. Experimentally, it has been shown that, with the ligand carrying a hydrogen atom at the P_1_′ site (glycine derivative **17a**), the pocket is virtually free of water molecules, even though it could size-wise accommodate up to five water molecules. Here, the binding of a ligand’s hydrophobic portions to the desolvated pocket results in a predominantly enthalpic response, as the cost of desolvation of the pocket must not be paid (see below).

Binding pockets generally exhibit a wide range of characteristics, from well-solvated pockets with ordered water molecules, as observed by crystallography, to virtually desolvated dry pockets. We therefore observe significant variation in the binding profile ranging from entropic to enthalpic dominance. This variation explains why the hydrophobic effect can thermodynamically shift from an entropic to an enthalpic signature.^46–53^

Another aspect is that residual mobility at the binding site can distinguish the thermodynamic profiles of very similar ligands. For example, two congeneric thrombin inhibitors that differ only in their terminal cyclopentyl or cyclohexyl substituents (**18** and **19**, Figure 2, *middle row, left*) exhibit different enthalpic and entropic binding signatures (ΔΔ*H* ^0^_**18⇒19**_ = 6.4 kJ/mol; −*T*ΔΔ*S* ^0^_**18⇒19**_ = −7.2 kJ/mol) when fitted into the S_3_/S_4_ pocket.^55^ The crystal structures reveal that the five-membered ring has well-defined difference electron density, while the six-membered analog remains undefined in the electron density due to pronounced disorder. MD simulations show that the cyclopentyl moiety undergoes pseudo- and jump-rotations, swapping its ring face. This motion leaves the electron density nearly unchanged, and the ligand experiences only minor changes in its interaction patterns with the protein. The cyclohexyl ring exhibits very different conformational properties even though the ring size increases by only one member. The MD trajectory demonstrates that the cyclohexyl moiety moves by exiting and reentering the pocket, which intermediately breaks a hydrogen bond. This explains the crystallographically observed disorder and the more entropically favored binding signature due to enhanced residual mobility of the ligand in the bound state.

Water can influence the profile even prior to protein binding. Among a series of protein kinase A (PKA) inhibitors (**20a**–**20e**, Figure 2, *middle row, right*), the ligand with the greatest number of rotatable bonds **20e** exhibits the most entropically favored signature, which appears counterintuitive.^56^ The opposite would be expected, since a more flexible ligand sacrifices a larger number of molecular degrees of freedom which is entropically unfavorable.^57^ A detailed analysis using nuclear magnetic resonance (NMR) and MD simulations revealed that, before binding to the protein, the most flexible ligand adopts conformational states (Figure 2 *middle row, far right*) in aqueous solution that tightly captures a water molecule. When this ligand binds to the protein, the fixed water molecule will be released. This release is entropically favorable and determines this ligand’s overall signature even though this step occurs before binding to the protein.

### Impact of Water on the Thermodynamic Binding Profile

The previous examples also highlight the important influences of water on ligand binding profiles.^58–62^ Interestingly, the water impact on binding affinity (ΔG^0^) is often minor. However, shifts in enthalpy/entropy compensation can be significant (see below). This may explain why docking and scoring are effective for predicting affinity even when the presence of water molecules is neglected. However, the presence of water in binding pockets will affect the geometry of the docked ligand binding poses, an aspect important to consider for correct design hypotheses.

Several examples were selected from the data in Figure 1a where the structural data suggest substantial impact of individual water molecules on the binding profile across pairs of congeneric ligands (Table S3). The data for 14 complex pairs are displayed in Figure 1b (right) in a ΔΔ*H* ^0^ versus −*T*ΔΔ*S* ^0^ diagram, depicting the relative difference of the pairs of complexes. Although differences in ΔΔG^0^ are usually small, in some cases contributions up to ±5 kJ/mol can be found. As for the complexes in Figure 1a, pronounced enthalpy/entropy compensation appears to be at work. A neutron diffraction study of five trypsin-ligand complexes showed that water molecules in the uncomplexed protein can either mediate interactions between protein and ligand in the formed complex, however, also some of them be displaced.^39,63^ Additionally, the remaining water molecules can change their binding properties with the accommodated ligand, transitioning from a fixed ordered to an enhanced dynamic state. These changes differ from one bound ligand to another. It will modulate the thermodynamic profile of the individual water molecules from a more enthalpic to a more entropic signature and vice versa. This fact will impact the comparison of the relative thermodynamic differences between individual binding profiles when a water molecule is displaced, picked up or spatially and dynamically shifted upon ligand binding. Therefore, a significant portion of the scatter found for enthalpy/entropy compensation in protein–ligand complexes likely results from the impact of water on the thermodynamic profile. As mentioned, the compensating effects for fragment binding are at least as significant as those for drug-size ligands (Figure 1a). Theoretically, fragments are usually assumed to be more enthalpy-driven binders because they bind to the hot spots in the binding pockets.^64,65^ However, due to their weak binding, the impact of water may be greater for fragments, which could explain the pronounced enthalpy/entropy compensation observed for their binding.

### Impact of Pocket Solvation on Ligand Binding Affinity

Our current knowledge of the thermodynamic profile of protein pocket solvation is still rather limited, primarily because accessing reliable enough experimental structural information and binding affinity data for individual water molecules in pockets is challenging and very difficult to collect. Therefore, we usually rely on computational simulation data.^66–69^ However, water molecules exhibit extensive dynamic behavior, and these properties change significantly with the presence of a bound ligand (see above^63^). The standard force fields implemented in our current MD simulations may not be reliable enough to describe all properties of water molecules, particularly in light of the huge dipole moment of water molecules and in consequence the induced polarization effects. An explicit handling of the water molecules is definitely required. Additionally, polarization phenomena depending on the local dielectric conditions (e.g., water molecules confined in a sealed pocket or in contact with the bulk water phase) must be considered, as well as the extensive establishment of hydrogen-bonding networks among water molecules, protein residues, and ligand functional groups.^70–73^ One cannot assume that the water density in an uncomplexed binding pocket is the same as or comparable to that of the surrounding bulk water phase, not to mention that water molecules can also adopt different protonation states in binding pockets.^74^ Furthermore, some pockets are transient and only open with ligand binding. What will their solvation patterns look like?

Stephen Homans first proposed the concept of pocket solvation with differing water densities prior to ligand binding.^75^ For enzymes, which must distinguish between different substrates, utilizing the solvation properties of a pocket is crucial for differentiating between correct and incorrect substrates. Thermolysin, an enzyme that recognizes peptide chains to be cleaved mainly in its deep S_1_′ pocket (its size would allow it to host up to five water molecules), provides an opportunity to study this phenomenon.^54^ Through high-resolution crystallography and microcalorimetry, we examined peptide-like phosphor-analog ligands (**17a**–**e**, Figure 2, *upper row, far right*). Conserved binding modes for all studied ligands were recorded. As already mentioned above, the ligand that orients only one hydrogen atom into the S_1_′ pocket has a *K*_i_ value that is 41,000 times lower than that of the derivative with a P_1_′ *sec*-butyl group. This side chain is structurally consistent with that of the natural substrate. How can a change of simply four carbon atoms with attached hydrogens result in such a tremendous affinity improvement, which is mainly of enthalpic origin? The first methyl group (H → CH_3_) exhibits one of the strongest “magic methyl group” effects known,^76–78^ and reveals an 140-fold improvement. Following an elaborate crystallization, phasing and refinement protocol, crystallographic analysis revealed that the S_1_′ pocket in thermolysin is virtually unsolvated.^54^ Therefore, a ligand to be bound does not incur the cost of desolvating the pocket. Interestingly, both a xenon atom and a benzene molecule can be hosted in the pocket.^54^ By crystallography, both probes indicate binding to the pocket, however their affinity to the pocket is most likely very low (Figure S2).

In another case we found a transient pocket in a tRNA-binding enzyme.^79^ Usually, this pocket remains closed, for example with ligand **21** (Figure 2, *bottom row, left*). However, the pocket opens through binding of a fragment (**22**) which itself does not even occupy the pocket. The crystal structure and MD simulations suggest that this pocket is well solvated with several water molecules. The binding of a designed ligand (**23**) that orients its hydrophobic propyn-1-yl side chain into the pocket replaces some water molecules but no improvement in binding affinity compared to the unsubstituted parent structure (**24**) is observed. Obviously, different to the thermolysin case,^21^ the occupancy of this pocket required significant desolvation costs and the placement of the hydrophobic substituent into this pocket reveals no affinity enhancement.

Additionally, the enzyme aldose reductase should be considered, which has a transient binding pocket competent to accommodate ligand side chains.^80,81^ This property allows the enzyme to recognize a wide variety of substrates, explaining its ability to degrade a broad range of structurally diverse aldehydes within the cell. Replacing a nitro group with a carboxylate group in the *meta*-position at the terminal end of a benzyl side chain yields two ligands (**25**, **26**) that differ by a surprising factor of 1,000 in binding affinity (Figure 3, *upper row*). Both ligands adopt different binding modes. The more potent nitro derivative **25** opens the transient pocket by inducing an intermediate peptide-bond flip in the protein. In contrast, the *meta*-carboxylate derivative **26** leaves the pocket closed and binds with a different orientation of the benzyl side chain outside the pocket. Initially, we attributed these differences to the high desolvation costs of the carboxylate group compared to the uncharged, isostructural nitro group. Modulating the properties of the benzyl substituent results in examples that leave the pocket either open or closed.^82^ Both binding modes were observed using crystallography and microcalorimetry.

**Figure 3:**
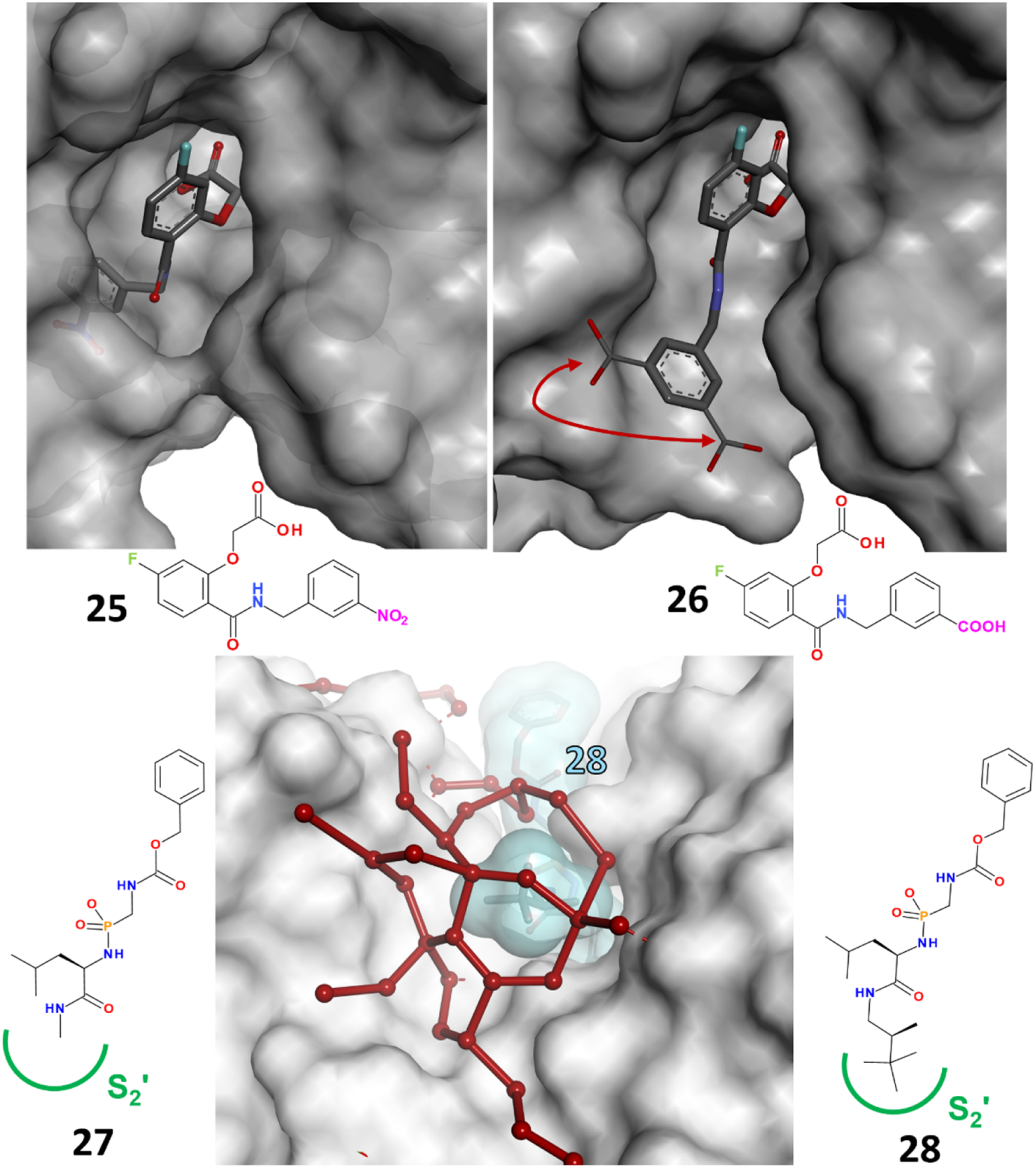
*Upper row*: Aldose reductase exhibits a transient pocket that opens upon binding to the nitro derivative **25**, which places its nitrophenyl moiety in the open pocket. The isosteric carboxy analog **26** binds outside the pocket with its terminal *meta*-carboxylate group disordered over two orientations; the pocket remains sealed in this case. Ligand **26** binds with a potency 1,000 times lower than that of ligand **25**. Mutations of residues involved in opening the transient pocket lead to a preflooding of the pocket. Consequently, the affinity advantage of the nitro derivative **25** over **26** is lost, and both ligands can bind to the open transient pocket. *Lower row*: The S_2_′ pocket of thermolysin is flat and bowl-shaped. Bound ligands remain partially exposed to the surrounding bulk water phase. Consequently, a new surface water network forms at the interface between the protein complex and the bulk water phase. Optimizing this network so that it assembles like a hood formed by polygons of hydrogen-bonded water molecules increased the binding affinity of **27** to **28** by a factor of 50. The image shows the water network around **28** as red spheres connected by hydrogen bonds, indicated by solid red lines. The protein (white) and ligand (cyan surface) are represented as in Figure 2.

For example, the unsubstituted benzyl derivative exhibits both open and closed states simultaneously.

The transient pocket is sealed by a hydrophobic phenyl-to-leucine side-chain contact, and opening the pocket involves flipping an adjacent peptide bond. To study the binding process to the transient pocket more closely, we replaced two leucine residues (positions 300, 301) in the crucial protein region with alanine residues.

The result was surprising.^81^ The 1,000-fold difference in affinity observed between the nitro and carboxylate derivatives in the wild type was reduced to a mere 10-fold difference in the examined mutated variants. Although the affinity of the nitro derivative **25** decreased significantly, it retains the binding mode observed in the wild type, occupying the open transient pocket. In contrast, the affinity of the carboxylate analog **26** changes only slightly between the wild type and the mutated variants. However, the binding mode with **25** switches from the geometry exclusively observed in the wild type with a closed transient pocket to a mixed situation in which both binding modes with closed and open pockets are observed. In the variant with both leucine residues replaced by alanine, the open transient binding pocket is exclusively found and occupied by the ligand. In all cases, opening the pocket involves flipping the peptide bond located upstream of amino acid 300.

Replacing Leu 300 with alanine creates additional volume that becomes occupied by several water molecules. This observation is supported by a crystal structure with citric acid as a ligand that fills the active site, but leaves the transient pocket unoccupied, while allowing water molecules to penetrate the structure. We also performed MD simulations and a hydration site analysis of the different pocket variants.^81^ According to these results, the transient pocket undergoes a “pre-flooding” in the variants, a phenomenon not observed in the wild type. This alters the desolvation balance, meaning the entering ligand must now displace water molecules. This gives the carboxylate derivative **26** an advantage because the “pre-flooded” water molecules can interact with the negatively charged carboxylate group, thereby stabilizing intermediate steps in the binding process. However, the uncharged nitro derivative **25** does not benefit from this stabilization. Instead, the “pre-flooding” is disadvantageous for the nitro derivative because it must also displace water molecules. This results in a significant decrease in affinity. This mechanism assumes, however, that the barrier to peptide flipping is significantly lower for the alanine variant than for the leucine derivative, or that it does not require a significant contribution.

To examine this effect, the glycine variant at position 300 was investigated as well.^83^ Because glycine has no side chain, it was expected to have a detectable influence on the peptide flip. The additional space generated by the smaller glycine side chain in the transient pocket should favor “pre-flooding”. The nitro derivative **25** binds to the open transient pocket of this variant and no peptide flip takes place during binding. The pocket is already sufficiently open due to the absence of a side chain. However, the binding affinity of the nitro derivative remains low. This underscores the fact that the peptide flip cannot exert a decisive influence on the binding affinity. Rather, the profile is largely determined by the transient pocket’s solvation properties when occupied. The carboxylate ligand **26** supports this assumption, as ligand binding now occurs exclusively into the open pocket when glycine is present in position 300.

What can we learn from these examples as a perspective for related cases with a transient pocket? Clearly, pocket desolvation significantly affects ligand binding affinity. The extent of this impact depends on how “pre-flooded” the pocket is before a ligand is accommodated. Pockets that are weakly or virtually unsolvated can increase binding affinity when occupied by hydrophobic portions of a ligand. The situation seems to be comparable for transient pockets that open only when a hydrophobic ligand portion approaches their entrance. Such processes are described as having an induced-fit binding mechanism.^84,85^ In this case, binding can occur without significant desolvation costs, and a strong affinity enhancement can be expected (cf. nitro derivative **25** in the wild type). However, structural or dynamic perturbations (which now favor a conformational selection binding mechanism) that lead to a “pre-flooding” of the transient pocket significantly reduce this enhancement because an increasing amount of free energy is required for pocket desolvation (cf. binding of **22** compared to **23** (TGT case) or **25** to **26** for the preflooded pocket). Finally, the question remains: How can hydrophobic, unsolvated binding pockets be detected? In crystallography, a well-known experimental phasing method is to pressurize protein crystals with noble gases.^86–88^ This method can also identify weakly solvated protein pockets (Figure S2). Benzene is another option because of its remarkable water solubility. However, benzene’s toxicity makes it hazardous to work with routinely. The Xe strategy worked well in thermolysin’s S_1_′ pocket and recently it helped to detect and fill a water-free void in the β1-adrenergic receptor.^89^

### Tailored Design of Surface-Water Networks around Hydrophobic Groups to Enhance Affinity

Finally, an additional pocket solvation effect can increase ligand binding affinity. Ligands usually occupy open, bowl or crevice-shaped binding pockets on the exterior of proteins. Often, a portion of the ligand extends into the surrounding bulk phase. However, at the newly formed interface of the protein–ligand complex to the surrounding bulk water phase, a novel solvation shell assembles.^90,91^ Depending on the structural features of the exposed ligand portion, the assembled interface will either allow for the formation of an ideal surface water network, or it will remain incomplete and perturbed due to a misfit with the geometry of the water shell. This will also impact ligand binding and in particular, the confinement of exposed hydrophobic groups by a perfect surface water network can significantly increase the affinity of the ligand. In a comprehensive study, we investigated the structural features of surface water networks formed around exposed protein–ligand interfaces next to the S_2_′ pocket of thermolysin.^92^ The formation of energetically favorable, fused, five- to eight-membered water polygons interconnected by chains of hydrogen bonds that wrap around the exposed ligand portion like a hood increases the ligand’s binding strength (Figure 3, *bottom row*). MD simulations can be used to design and validate suitable side chains by considering only the mobility of the water molecules.^93^ Using this design concept, we achieved a 50-fold affinity enhancement of **27** over **28**. Additional examples considering the formation of water networks between protein and ligand have been described.^94,95^ However, caution is required when applying this concept to polar or charged groups that extend beyond the binding pocket and which were intentionally added to improve ligand solubility without altering its binding pose. The formation of the polygonal water hood can be strongly perturbed by such groups, thereby nullifying the enhancing water shell effect.^96^

## Conclusions

Of the four fundamental forces of physics,^97^ biomolecules use almost exclusively the electromagnetic forces to interact, and all processes constituting life result from these interactions. Molecules can only interact; however, they do this in a highly sophisticated manner with astonishing diversity and incredible complexity. Therefore, we use terms such as *molecular recognition*, *functional steering*, *information transfer*, or *signal transduction*, to name a few, in a figurative sense to conceptualize the observed effects at a higher level. Although thermodynamics can characterize interactions more precisely and differentiate in terms of enthalpy and entropy, we can only determine them as general overall quantities, valid for the entire multicomponent biological systems. The problem of partitioning these overall interaction quantities into separate contributions assigned to individual components of the system then arises. This can only be achieved by collecting as much structural background information as possible about the system by studying related ligand pairs where only one property will hopefully dominate the profile change. In drug design, the property of interest is the Gibbs free energy of binding (affinity), which results from a complex sum of many enthalpy and entropy contributions. Many of these contributions cancel each other out due to compensating enthalpy and entropy effects. Therefore, they do not appear in the measured Gibbs free energy. Nevertheless, a few contributions remain as determining factors, but, they can vary greatly from case to case. This makes it difficult to use thermodynamic data as a straightforward guideline for drug optimization. Nevertheless, some general rules can be suggested and have been summarized in detail elsewhere.^39^

In this perspective, it has been attempted to demonstrate which aspects determine a given thermodynamic enthalpy/entropy signature by examining selected examples of closely related ligand pairs. Apart from significant compensating effects, determinants include correct rigidification, burial of strong polar interactions involving charges, residual mobility at the protein binding site, analysis of the ligand’s conformational properties prior to protein binding, and the impact of ubiquitously present water molecules as third binding partner in protein–ligand complex formation. The presence or absence of water molecules, along with their structural characteristics, significantly impact the ligand binding signature. A substantial part of enthalpy/entropy compensation observed in protein–ligand complex formation is likely due to the contributions of water molecules. Nevertheless, once recognized, the properties of the participating water molecules can be exploited in the design concept to improve a given ligand’s binding affinity.

By the end of this article, perhaps practical medicinal chemists will ask themselves: “How can I apply all these wonderful insights? Are the thermodynamic data reflecting the complexity of the phenomena actually useful for my work?” First, it is helpful to understand which factors can contribute to affinity enhancement. There is more involved than simply counting the number of hydrogen bonds formed, the amount of hydrophobic surface buried in a binding pocket, or the number of water molecules displaced from a pocket. Differences in ligand properties prior to protein binding can determine affinity. Rigidification of a ligand only contributes if the bound ligand geometry is already present in solution prior to protein binding. Differences in pocket solvation must also be considered. More aspects have been summarized elsewhere.^39^

But second, can thermodynamic data help guide lead optimization campaigns in a standard medicinal chemistry setting? In the pharmaceutical industry, collecting ITC data across a series of related protein–ligand complexes is usually not a significant undertaking. If one complex in such a study falls outside a trend, it is likely a case where other aspects matter and analyzing the example in more detail is worthwhile. One possible reason is changes in protonation state. This involves estimating p*K*_a_ values, particularly with regard to the composition of the target’s binding pocket. For example, are acidic and basic residues or functional groups present? Medicinal chemists are usually very well trained in estimating putatively induced ligand p*K*_a_ shifts (e.g., assumed isosteric replacement of heterocycles).

However, other reasons may explain why a ligand falls outside the series. Two examples presented in this contribution should illustrate the perspective of this concept. The intramolecular rigidification to preorganize a ligand (**13** ⇒ **14, 15**, Figure 2, *upper row, center left*) in solution prior to protein binding to thrombin clearly shows differences in thermodynamic profiles and indicate differences in ligand behavior that matter for optimization strategies. Another example is the paradoxical correlation that the most flexible ligand in the PKA series (**20a**–**e**, Figure 2, *middle row, right*) is the most entropic binder. Collecting ITC data on such a complex series would make researchers aware of other important factors resulting from effects prior to protein binding. These examples demonstrate that collecting thermodynamic data, which is achievable in an industrial setting, can help explain seemingly unexplainable trends in a ligand series’ structure–activity data. In this way, thermodynamic data can complement a drug optimization strategy.

## Supplementary Material



## Notes

Thermodynamic data and PDB-code references of the 266 enzyme-ligand complexes, 55 endothiapepsin-fragment complexes, and thermodynamic difference data of the 14 ligand pairs associated with a pick-up/release of water molecules; Figure S1 with Δ*H* ^0^ versus *–T*Δ*S* ^0^ plots of the ITC data of the 266 protein–ligand complexes, color-coded for the seven different enzymes; Figure S2 of the S_1_′ pocket of thermolysin with bound Xe and benzene (PDF)

## Abbreviations

Δ*G* ^0^: Gibbs free energy
Δ*H* ^0^: enthalpy
*–T*Δ*S* ^0^: entropy multiplied by the absolute temperature in Kelvin
AR: aldose reductase
CA II: human carbonic anhydrase
*IC*_50_: half-maximal inhibitory concentration
ITC: isothermal titration Calorimetry
*K*_i_, *K*_d_, or *K*_a_: equilibrium inhibition constant, dissociation constant, association constant
MD: molecular dymanics
NMR: nuclear magnetic Resonance
TGT: tRNA guanine transglycosylase
