# Supplementary material for: Thermodynamic Data Remain a Hot Tip for Decoding Binding Affinity and Water Impact on Protein–Ligand Complex Formation to Assist Lead Optimization
Source: J Med Chem. 2026 Feb 17;69(4):3719–30. doi: 10.1021/acs.jmedchem.5c03100 (PMC12951438; doi:10.1021/acs.jmedchem.5c03100)
Supplement: Supplementary file 1 [file jm5c03100_si_001.pdf]

Supporting Information

Thermodynamic Data Remain a Hot Tip for Decoding Binding Affinity and Water Impact on Protein-Ligand Complex Formation to Assist Lead Optimization

Gerhard Klebe, Institute of Pharmaceutical Chemistry, Philipps University Marburg, Marbacher Weg 6, 35032 Marburg, Germany, Email: Klebe@mailer.uni-marburg.de*

Contents of Supporting Information

Table S1: Excel-Table with the thermodynamic data of 266 complexes taken from literature (***Page S2-S8***)

Table S2: Excel-Table with the thermodynamic data of 55 endothiapepsin-fragment complexes (***Page S9-S11***)

Table S3: Excel-Table with the thermodynamic difference data of 14 pairs of closely related complexes where ligand binding is mainly determined by the pick-up or release of water molecules (***Page S12***)

Figure S1 (***Page S13***)

Figure S2 (***Page S14***)

A	B	C	D	E	F	G	H
Journal	Link to DOI	No.	ΔG	ΔH	- TΔS	Target	PDB entry
JMC	https://pubs.acs.org/doi/10.1021/acs.jmedchem.9b01196	1	-39.1	-55.3	16.2	Thrombin	5JZY
		2	-37.3	-52.6	15.3	Thrombin	5LCE
		3	-45.6	-54.6	8.4	Thrombin	5LPD
		4	-31.4	-37.1	5.7	Thrombin	6ROT
		5	-43.5	-52.1	8.6	Thrombin	3RML
		6	-38.9	-37.4	-1.4	Thrombin	6GBW
		7	-44	-60.9	16.9	Thrombin	5JFD
CMC	https://doi.org/10.1002/cmdc.202300222	1	-44.3	-65.6	21.3	AR	4QBX
		2	-25.4	-36.4	11	AR	4YS1
		1	-32.3	-63.6	31.3	AR L300A	6T3P
		2	-26.1	-38.7	12.6	AR L300A	6TXP
		1	-29.3	-100.7	71.4	AR L301A	6TD8
		2	-23.9	-48.3	24.5	AR L301A	6T7Q
		1	-30.3	-82.7	52.4	AR L300/301A	6XUM
		2	-25.1	-60.3	35.2	AR L300/301A	6Y1P
Biomolec	https://doi.org/10.3390/biom11121837	3	-30.3	-39.1	8.8	AR	6TUF
		4	-32.2	-45	12.8	AR	6TUC
		5	-23.4	-30	6.6	AR	n.d.
		6	-22.6	-37.5	14.9	AR	6SYW
		7	-29.6	-35.4	5.8	AR	4PUU
		8	-29.3	-29.9	0.6	AR	4Q7B
JMC	https://dx.doi.org/10.1021/acs.jmedchem.0c01809	1L	-45.2	-38.8	-6.4	Thrombin	5MM6
		2L	-42.9	-39.5	-3.4	Thrombin	6T57
		3L	-34.8	-19.2	-15.6	Thrombin	6T53
		1L	-42.6	-38.2	-4.4	Trypsin	6ZQ2
		2L	-31.1	-21.8	-9.3	Trypsin	5MNQ
		3L	-33.4	-28.9	-4.6	Trypsin	6YDY
Biomolec	https://dx.doi.org/10.3390/biom10040509	1	-36	-39.6	3.6	CAII	6GDC
		2	-38	-40.8	2.8	CAII	6GM9
		3	-38.2	-48.2	9.9	CAII	6RIT
		4	-40.2	-47.4	7.2	CAII	6RQI
		5	-37.3	-39.8	2.5	CAII	6RKN
		6	-42.1	-42.7	0.6	CAII	6RJJ
		7	-42.8	-54.4	11.7	CAII	6RNP
		8	-38.3	-42.3	4	CAII	6ROE
		9	-40.9	-41.9	1	CAII	6RRG
		10	-42.6	-44.2	1.6	CAII	6RRI
		11	-45.5	-44	-1.5	CAII	6RS5
		12	-48.6	-42.3	-6.3	CAII	6RSZ
		13	-48.1	-39.5	-8.5	CAII	6S9G
		15	-41.1	-43.8	2.7	CAII	6ROB
		16	-41.8	-45.2	3.4	CAII	6RH4
		17	-31.3	-44.8	13.5	CAII	6RL9
JMC	https://dx.doi.org/10.1021/acs.jmedchem.9b02061	1	-39.8	-109.4	69.6	Thrombin	6HSX
		1	-23.8	-56.2	32.4	Trypsin	6T0M

		2	-34.4	-86	51.6	Thrombin	6T3Q
		2	-25.3	-56.1	30.8	Trypsin	6T3Q
		3	-36.5	-98.2	61.7	Thrombin	6T4A
		3	-24.9	-53.6	28.7	Trypsin	6T5W
		4	-29.4	-30.6	1.2	Thrombin	6TDT
		4	-17.7	-61.9	44.3	Trypsin	6SY3
ACS CB	https://dx.doi.org/10.1021/acschembio.9b00895	1a	-36	-39.6	3.6	CAII	6GDC
		1b	-38	-40.8	2.8	CAII	6GM9
		1c	-40.4	-41.5	1.1	CAII	6HQX
		1d	-40.7	-38.6	-2.1	CAII	6HR3
		1e	-42.6	-44.2	1.6	CAII	6HXD
		1f	-45	-43	-2	CAII	6SBH
		2a	-37.3	-45.5	8.2	CAII	6I0W
		2b	-39.5	-46.5	7	CAII	6I1U
		2c	-42.6	-49.8	7.2	CAII	6I2F
		2d	-43.6	-51.8	8.3	CAII	6I3E
ACS CB	https://doi.org/10.1021/acschembio.9b00476	1	-37.3	-26.2	-11.1	PKA	5M6V
		2	-39.2	-39.1	-0.1	PKA	5M6Y
		3	-35.6	-29	-6.5	PKA	5M75
		4	-32.6	-28.2	-4.4	PKA	5M71
		5	-36.1	-33.1	-3.1	PKA	5LCP
JMC	https://doi.org/10.1021/acs.jmedchem.8b00105	01 -PKI	-31.3	-35.7	4.3	PKA	5LCU
		02 -PKI	-31.9	-37	5.1	PKA	5LCT
		Fasudil - PKI	-36.1	-33.1	-3.1	PKA	5LCP
		04 -PKI	-35.4	-35.1	-0.3	PKA	5LCR
		05 -PKI	-32	-22.8	-9.2	PKA	5LCQ
		01 +PKI	-28.5	-16.2	-12.3	PKA	5LCU
		02 + PKI	-29.9	-17.9	-12	PKA	5LCT
		Fasudil+PKI	-32.8	-15.8	-17	PKA	5LCP
		04 +PKI	-32.6	-15.7	-16.9	PKA	5LCR
		05 + PKI	-31.5	-12.5	-19.1	PKA	5LCQ
CMC	https://doi.org/10.1002/cmdc.201800438	1	-29.7	-38.7	9.1	PKA	5M0C
		2	-27.9	-35.1	7.2	PKA	5M0L
		3	-35.4	-31.8	-3.5	PKA	5M0B
		4	-32	-22.8	-9.2	PKA	5LCQ
NatC	http://dx.doi.org/10.1038/s41467-018-05769-2	N-amidino-piperidine	-22	-3.8	-18.2	Trypsin	5MO2
		N-amidino-piperidine	-21.3	-6.9	-14.4	Trypsin	5MNG
		N-amidino-piperidine	-20.8	-12.9	-7.9	Trypsin	5MNO
		Benzamidine	-26.7	-11.7	-15	Trypsin	5MO0
		Benzamidine	-26.5	-15.9	-10.6	Trypsin	5MNG
		Benzamidine	-26.5	-19.9	-6.6	Trypsin	5MNF
JACS	http://dx.doi.org/10.1021/jacs.7b05028	2	-25.1	-22.7	-2.4	Thermolysin	3FVP
		3	-34.3	-33.7	-0.5	Thermolysin	3FLF
		4	-39.1	-45.1	5.9	Thermolysin	4H57
Diss Fokkens S 79, 107		CRC220	-49.2	-48.5	-0.7	Thrombin	1YPK
		1 obst	-42.3	-6.19	-36.1	Thrombin	1YPE
		5 obst	-31.9	-7.3	-24.6	Thrombin	1YPG
		1 obst	-30.5	0.03	-30.5	Trypsin	1Y3U
		2 obst	-25.7	-4.5	-21.2	Trypsin	1Y3V

			3 obst	-25.7	-5.23	-20.5	Trypsin	1Y3W
			5 obst	-29.9	-6.45	-23.5	Trypsin	
			6 obst	-25.8	-1.66	-24.1	Trypsin	1Y3X
JMC	http://dx.doi.org/10.1021/jm5006868		1a	-41.6	-97.4	55.8	TGT	4PUK
			1b	-36.5	-47.7	11.2	TGT	3S1G
			2a	-42.6	-96.4	53.8	TGT	4PUJ
			2b	-36.7	-48.9	12.2	TGT	4Q4R
			3a	-42.6	-78.9	36.3	TGT	4Q4O
			3b	-36.5	-50.6	14.1	TGT	4Q4P
			4a	-39.7	-66.3	26.6	TGT	4Q4S
			4b	-33	-42.1	9.1	TGT	4Q4Q
JMC	http://dx.doi.org/10.1021/jm500401x		1	-42.6	-96.4	53.8	TGT	4PUJ
			1	-33.8	-66.2	32.4	TGTE235Q	
			2	-41.6	-97.4	55.8	TGT	4PUK
			2	-39	-33.9	-4.9	TGTD102N	4PUL
			2	-38.5	-53.2	12.6	TGTD156N	4PUM
			3	-36.5	-48.4	11.9	TGT	3S1G
			4	-37.4	-89.2	51.8	TGT	2BBF
BOMC	http://dx.doi.org/10.1016/j.bmc.2016.07.053		1	-41.6	-74.8	33.2	TGT	1PUK
			2	-39.7	-50.9	11.2	TGT	4Q4S
			3	-42.6	-66.7	24.1	TGT	4Q4R
			4	-42.6	-78.3	35.7	TGT	4Q4O
			5	-41.4	-45.3	3.9	TGT	4Q8T
			6d	-43	-47	4	TGT	4Q8W
JMC	http://dx.doi.org/10.1021/acs.jmedchem.5b00812		A	-37.5	-45.1	7.6	Thrombin	4UDW
			B	-39.7	-28.8	-10.9	Thrombin	2ZGX
			C	-38.8	-20.7	-18.1	Thrombin	5AFZ
			1	-17.2	-15.7	-1.5	Thrombin	5AF9
			2	-19	-29.7	10.7	Thrombin	4UD9
			3	-19.8	-13.9	-5.9	Thrombin	4UEH
			4	-22.8	-15.4	-7.4	Thrombin	4UE7
CMC	http://dx.doi.org/10.1002/cmdc.201500531		2	-43.8	-17	-26.8	Thrombin	4UFE
			3	-44.4	-23.1	-21.3	Thrombin	4UFF
			4	-43.1	-13.9	-29.2	Thrombin	4UFG
			5	-53.9	-16.9	-37	Thrombin	4UFD
JMC	http://dx.doi.org/10.1021/acs.jmedchem.7b00490		1	-29.6	-29.4	-0.2	Thermolysin	5N2Z
			2	-28.4	-30.9	2.6	Thermolysin	2N2T
			3	-26.2	-30.1	3.9	Thermolysin	5N34
			4	-25.3	-17.2	-8.2	Thermolysin	5N3V
			5	-32.8	-38.1	5.3	Thermolysin	5N31
			6	-33.7	-37.5	3.8	Thermolysin	5N2X
			7	-30	-32.4	2.4	Thermolysin	5MNR
			8	-28.5	-37.5	9	Thermolysin	5N3Y
			9	-36.1	-41.1	5	Thermolysin	4MXJ
			10	-32.8	-33.2	0.4	Thermolysin	3T8F
			11	-40.1	-51.2	11.1	Thermolysin	3FVP
			12	-40.6	-54	13.4	Thermolysin	3ELF
ACS CB	http://dx.doi.org/10.1021/acschembio.7b00062		4	-29.6	-35.4	5.8	AR	4PUU
			5	-31.5	-46.2	14.7	AR	4PUW

		6	-29.3	-29.9	0.6	AR	4Q7B
		7	-41.5	-54	12.5	AR	4QR6
		8	-44.3	-65.6	21.3	AR	4QBX
		9	-25.4	-36.4	11	AR	4YS1
		11	-43.5	-55.8	12.3	AR	4RPQ
JMC	http://dx.doi.org/10.1021/acs.jmedchem.6b00998	1	-36.8	-23	-13.8	Thermolysin	4MTW
		2	-37.9	-21.3	-16.6	Thermolysin	5JT9
		3	-38.5	-21.2	-17.2	Thermolysin	5Js3
		4	-36.6	-18.5	-18.1	Thermolysin	5JXN
		5	-37.2	-16.9	-20.3	Thermolysin	5JVI
		6	-36.8	-15	-21.8	Thermolysin	5JSS
CMC	http://dx.doi.org/10.1002/cmdc.201500393	3	-40.4	-59.7	19.3	AR	4XZH
BBA	http://dx.doi.org/10.1016/j.bbagen.2014.12.007	A	-37.5	-45.1	7.6	Thrombin	4UDW
		B	-39.7	-28.8	-10.9	Thrombin	2ZGX
		C	-38.8	-20.7	-18.1	Thrombin	5AFZ
		1	-17.1	-15.7	-1.5	Thrombin	5AFF9
		2	-19	-29.7	10.7	Thrombin	4UD9
		3	-19.8	-13.9	-5.9	Thrombin	4UEH
		4	-22.9	-15.4	-7.4	Thrombin	4UE7
CMC	http://dx.doi.org/10.1002/cmdc.201400013	1	-31.8	-15.5	-16.3	Thermolysin	3T73
		2	-33.7	-20.6	-13.1	Thermolysin	3T8F
		3	-37.5	-26.7	-10.8	Thermolysin	4MXJ
		4	-37.9	-21.4	-16.5	Thermolysin	4MTW
		5	-38.8	-22.3	-16.6	Thermolysin	4MZN
		6	-36	-16.4	-19.6	Thermolysin	4N5P
		7	-36.4	-14.6	-21.8	Thermolysin	4N4E
		8	-34.4	-11.5	-22.9	Thermolysin	4N66
		9	-33.8	-10.9	-22.9	Thermolysin	4MWP
AC	http://dx.doi.org/10.1002/ange.201208561	1	-32.8	-19	-13.8	Thermolysin	3T8G
		2	-38.5	-32.4	-6.1	Thermolysin	3T74
		3	-40.7	-33.6	-7.1	Thermolysin	3T87
		4	-40.6	-31	-9.6	Thermolysin	3T8H
		5	-40.6	-30.4	-10.2	Thermolysin	3T8C
		6	-38.7	-22.7	-16	Thermolysin	3T8D
		7	-39.4	-21.8	-17.6	Thermolysin	4H57
		8	-36.5	-18.3	-18.2	Thermolysin	4D9W
JMC	http://dx.doi.org/10.1021/jm300337q	1 ACB	-47.8	-35.4	-12.4	Thrombin	3RML
		2 ACB	-47.2	-32.1	-15.1	Thrombin	3RMM
		3 ACB	-54.2	-36.2	-18	Thrombin	3RMN
		4 ACB	-54.3	-30.1	-24.2	Thrombin	3T5F
		5 ACB	-54.5	-28.7	-25.8	Thrombin	3RMO
		6 AMBA	-44.9	-14.3	-30.6	Thrombin	3RLW
		7 AMBA	-42.6	-15.9	-26.7	Thrombin	3RLY
		8 AMBA	-47.1	-13.8	-33.3	Thrombin	3RM0
		9 AMBA	-51.9	-10.4	-41.5	Thrombin	3UWJ
		10 AMBA	-53.7	-11.4	-42.3	Thrombin	3RM2
JMB	http://dx.doi.org/10.1016/j.jmb.2012.01.054	1 Phenyl	-31.7	-13.6	-18.1	Thrombin	2ZFF
		2 mPy	-25.8	-17.7	-8.1	Thrombin	3P17
		3 mPyMe+	-23.9	-18.7	-5.2	Thrombin	3QTO

		4 o Py	-25	-19.8	-5.2	Thrombin	3SI3
		5 oPyMe+	-21.4	-15.5	-5.9	Thrombin	3SI4
		6 pPy	-24.2	-18.7	-5.5	Thrombin	3SV2
		7 pPyMe+	-24	-23.6	-0.4	Thrombin	3QTV
		8 mCloPy	-31.5	-30.3	-1.2	Thrombin	3SHC
		9 mCloPyMe+	-23.7	-23.3	-0.4	Thrombin	3QX5
		11 oClmPyMe+	-24.3	-22.4	-1.9	Thrombin	3QWC
		12 AMBA	-47.3	-40.2	-7.1	Thrombin	
		13 o,mDiCl	-37.6	-34.5	-3.1	Thrombin	
JMB	http://dx.doi.org/10.1016/j.jmb.2009.04.051	1a mF	-31.3	-13.1	-18.2	Thrombin	2ZDV
		4 Benzamidine	-46.1	-40.1	-6.1	Thrombin	2ZDA
		6a o,m,Di Cl	-38.4	-41.3	2.9	Thrombin	
		6e oF mCl	-37.3	-41	3.8	Thrombin	
		6b oMe mCl	-37.2	-33.5	-3.7	Thrombin	
		1c mBr	-35.8	-34.5	-1.3	Thrombin	
		1b mCl	-35.4	-37.1	1.7	Thrombin	2ZC9
		3a mMe	-34.8	-28.5	-6.3	Thrombin	2ZF0
		1d mIod	-34.5	-38	3.5	Thrombin	
		7a mNH2	-34.4	-31.9	-2.5	Thrombin	
		3b mEt	-32.9	-16.5	-16.4	Thrombin	
JMB	http://dx.doi.org/10.1016/j.jmb.2009.06.016	1	-31.3	-33.5	2.2	Thrombin	2ZFP
		2	-35.4	-37.1	1.7	Thrombin	2ZC9
		3	-39.6	-45.6	6	Thrombin	3DHK
		4	-40.1	-38.7	-1.4	Thrombin	2ZGX
		5	-46.1	-40.1	-6.1	Thrombin	2ZDA
		6	-48.5	-47.5	-0.9	Thrombin	2ZO3
		7	-38.9	-35.6	-3.4	Thrombin	3DUX
		8	-32.8	-19.4	-13.2	Thrombin	3F68
JMB	http://dx.doi.org/10.1016/j.jmb.2010.02.007	2c	-31.3	-33.5	2.2	Thrombin	2ZFP
		2e	-35.1	-42.7	7.5	Thrombin	2ZGB
		2l	-35.4	-37.1	1.7	Thrombin	2ZC9
		3c	-32.8	-27	-5.8	Thrombin	2ZI2
		3e	-37.4	-22.7	-14.7	Thrombin	2ZIQ
		3l	-37.8	-28.6	-9.3	Thrombin	2ZHQ
		4c	-40.1	-38.7	-1.4	Thrombin	2ZGX
		4e	-42.9	-34.5	-8.4	Thrombin	2ZNK
		4l	-46.1	-40.1	-6.1	Thrombin	2ZDA
		3c	-32.8	-16.2	-16.6	Thrombin	2ZI2
		3e	-37.4	-11.9	-25.5	Thrombin	2ZIQ
		3l	-37.8	-17.8	-20	Thrombin	2ZHQ
JMB	http://dx.doi.org/10.1016/j.jmb.2008.03.063	1	-46	-58.1	12.1	AR WT	2FZ8
		2	-42.5	-48.5	6	AR WT	2PDG
		3	-42.7	-59	16.3	AR WT	2IKI
		4	-42.2	-81.2	39	AR WT	2IKJ
		5	-37.9	-54.7	16.8	AR WT	1AH0
		6	-46.7	-79.5	32.8	AR WT	1PWM
		1	-42.4	-51.6	9.2	AR V47I	2PD5
		6	-46.1	-84.3	38.2	AR V47I	2PD9
		1	-40	-42.1	2.1	AR F121P	2PDB

		4	-37.6	-66.2	28.6	AR F121P	2PDC
		1	-38.2	-75.7	37.5	AR L300P	2PDF
		2	-37.9	-57.3	19.4	AR L300P	2PDH
		1	-41.5	-58.4	16.9	AR L300A	2PDI
		4	-40.3	-61.9	21.6	AR L300A	2PDJ
		1	-36.5	-81.1	44.6	AR S302R	2PDM
		2	-30.9	-68	37.1	AR S302R	2PDN
		4	-31.7	-61.8	30.1	AR S302R	2PDP
		2	-32.5	-37.7	5.2	AR C303D	2PDQ
		4	-31.9	-80.5	48.6	AR C303D	2PDU
		6	-42.4	-84.7	42.3	AR C303D	2PDW
		1	-32.1	-81.9	49.8	S302R C303D	2PDX
		6	-38.8	-76.5	37.7	R S302 C303D	2PDY
		5	-37.5	-54.4	16.9	AR L301M	2PDK
		7	-35.5	-32.3	-3.2	AR L301M	2PDL
AgCh	http://dx.doi.org/10.1002/anie.200701169	1a cPent	-35.4	-16.9	-18.5	Thrombin	3LJJ
		1b cHex	-36.2	-10.5	-25.7	Thrombin	3LJO
JMB	http://dx.doi.org/10.1016/j.jmb.2006.12.004	1	-35.4	-25.6	-9.8	AR	2IKG
		2	-31.3	-8.7	-22.6	AR	2IKH
		3	-42.7	-62.1	19.4	AR	2IKI
		4	-42.2	-79.1	36.9	AR	2IKJ
JMC	http://dx.doi.org/10.1021/jm050424+	Sorbinil	-37.9	-51.8	13.9	AR	1AH0
		Sorbinil	-38.7	-46.7	8	AR L300P	
		Fidarestat	-46.7	-75.5	28.8	AR	1PWM
		Fidarestat	-38.9	-68.6	29.7	AR L300P	2AGT

Table S1: Excel-Table with the thermodynamic data of 266 complexes taken from literature (full listing, see deposited Table S1.xlcx)

Column A: abbreviation for journal
Column B: DOI link to corresponding papers
Column C: Running No. of ligand in the paper
Column D: ΔG in kJ/mol
Column E: ΔH in kJ/mol
Column F: -TΔS in kJ/mol
Column G: Target (no. of entries): Aldose Reductase (AR, 54), human carbonic anhydrase (CAII, 26), protein kinase A (PKA 19), Thermolysin (38), Thrombin (90), Trypsin (18), tRNA guanine transglycosylase (TGT, 21)
Column H: PDB-code of corresponding crystal structure

Reference links to the considered publications:

https://pubs.acs.org/doi/10.1021/acs.jmedchem.9b01196
https://doi.org/10.1002/cmdc.202300222

https://doi.org/10.3390/biom11121837
https://dx.doi.org/10.1021/acs.jmedchem.0c01809
https://dx.doi.org/10.3390/biom10040509
https://dx.doi.org/10.1021/acs.jmedchem.9b02061
https://dx.doi.org/10.1021/acschembio.9b00895
https://doi.org/10.1021/acschembio.9b00476
https://doi.org/10.1021/acs.jmedchem.8b00105
https://doi.org/10.1002/cmdc.201800438
http://dx.doi.org/10.1038/s41467-018-05769-2
http://dx.doi.org/10.1021/jacs.7b05028
http://dx.doi.org/10.1021/jm5006868
http://dx.doi.org/10.1021/jm500401x
http://dx.doi.org/10.1016/j.bmc.2016.07.053
http://dx.doi.org/10.1021/acs.jmedchem.5b00812
http://dx.doi.org/10.1002/cmdc.201500531
http://dx.doi.org/10.1021/acs.jmedchem.7b00490
http://dx.doi.org/10.1021/acschembio.7b00062
http://dx.doi.org/10.1021/acs.jmedchem.6b00998
http://dx.doi.org/10.1002/cmdc.201500393
http://dx.doi.org/10.1016/j.bbagen.2014.12.007
http://dx.doi.org/10.1002/cmdc.201400013
http://dx.doi.org/10.1002/ange.201208561
http://dx.doi.org/10.1021/jm300337q
http://dx.doi.org/10.1016/j.jmb.2012.01.054
http://dx.doi.org/10.1016/j.jmb.2009.04.051
http://dx.doi.org/10.1016/j.jmb.2009.06.016
http://dx.doi.org/10.1016/j.jmb.2010.02.007
http://dx.doi.org/10.1016/j.jmb.2008.03.063
http://dx.doi.org/10.1002/anie.200701169
http://dx.doi.org/10.1016/j.jmb.2006.12.004
http://dx.doi.org/10.1021/jm050424+

A	B	C	D	E
Frag. No.	ΔG	ΔH	-TΔS	https://doi.org/10.2210/pdb????/pdb
F004	-16.30	7.90	–24,20	4Y36
F005	-21.70	-23.20	1.50	4Y3E
F014	-11.00	-14.20	3.20	4Y4U
F017	-14.90	-44.70	29.80	4Y3P
F035	-12.80	-44.80	32.00	4Y3W
F039	-13.40	-22.90	9.50	5OZS
F041	-20.70	-30.10	9.40	4Y3Z
F042	-14.90	-18.80	3.90	4Y43
F051	-15.00	-81.20	66.20	4Y4X
F052	-13.50	-34.00	20.50	4Y51
F054	-21.20	-21.60	0.40	4Y53
F056	-11.50	-20.40	8.90	4Y54
F058	-11.00	-53.20	42.20	4Y56
F063	-18.00	-54.80	36.80	4Y57
F073	-11.40	-10.10	-1.30	4Y4Z
F075	-24.50	-20.70	-3.80	4Y39
F078	-14.00	-21.80	7.70	4Y3F
F081	-13.90	-25.00	11.20	4Y50
F103	-13.30	-20.10	6.80	4Y3M
F112	-16.50	-53.70	37.20	4Y41
F114	-18.70	-33.30	14.50	4Y4T
F125	-14.90	-29.70	14.70	4Y4W
F131	-18.80	16.90	-35.70	4Y4E
F158	-15.90	-25.60	9.60	5J25
F162	-18.00	-38.80	20.80	4Y47
F164	-20.30	-1.80	-18.60	4Y44
F171	-12.90	-49.60	36.70	4Y3X
F189	-15.00	-14.20	-0.70	4Y3H
F205	-17.20	-5.40	-11.70	4Y3L
F206	-11.40	-53.60	42.10	4Y5A

F207	-16.20	-5.60	-10.60	4Y3T
F211	-15.70	-23.30	7.70	4YCK
F216	-16.30	-38.30	21.90	4YCT
F224	-20.20	-16.70	-3.50	4YD3
F227	-12.30	-24.20	11.90	4YD4
F231	-11.80	-41.90	30.10	5DR4
F236	-17.20	-19.60	2.50	4Y5D
F240	-12.90	-18.70	5.70	4YD6
F260	-12.60	-34.70	22.10	4Y58
F261	-17.40	-30.20	12.70	4Y5B
F266	-12.40	-6.10	-6.40	4Y36
F267	-16.70	-43.90	27.20	4Y5C
F268	-11.60	-58.10	46.50	4Y5E
F272	-17.80	-64.00	46.20	4Y5G
F273	-11.50	-32.50	21.00	4Y3N
F274	-12.00	-89.00	77.20	4Y5K
F284	-20.40	-38.40	18.00	5P6N
F285	-13.00	-27.00	14.00	4Y3G
F286	-13.20	-77.40	64.30	4Y4A
F291	-17.70	-27.90	10.20	4Y45
F321	-15.00	-13.60	-1.40	4Y3D
F323	-18.80	-8.50	-10.30	4Y4B
F328	-21.00	5.50	-26.50	4Y5M
F337	-19.20	-9.10	-10.10	4Y5N
F338	-19.10	-30.90	11.90	4Y5P
SAP114	-37.40	-50.40	13.00	4LAP

Table S2: Excel-Table with the thermodynamic data of 55 endothiapepsin-fragment complexes taken from Wang. [Wang, Xiaojie. Characterization of fragment binding to endothiapepsin studied by crystallography and calorimetry. Master thesis, Dept. of Chemistry, Univ. of Marburg (2015)].

Column A: Label of the fragment according to the numbering in Radeva, N. et al. Active Site Mapping of an Aspartic Protease by Multiple Fragment Crystal Structures: Versatile Warheads to Address a Catalytic Dyad. J. Med. Chem. 59 (2016) 9743–9759. http://dx.doi.org/10.1021/acs.jmedchem.6b01195. and Radeva, N. et al. Remote Interplay of Small Molecules with Endothiapepsin – Hot spot analysis. J. Med. Chem. 59 (2016). 7561–7575. http://dx.doi.org/10.1021/acs.jmedchem.6b00645

Column B: ΔG in kJ/mol
Column C: ΔH in kJ/mol
Column D: -TΔS in kJ/mol
Column E: pdb-code of the corresponding crystal structure (accessible via https://doi.org/10.2210/pdb????/pdb where ???? stands for the four letter pdb-code)
Final row: SAP114 reference ligand used for displacement titrations

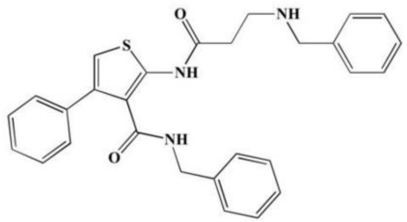

SAP114 was used as reference ligand in all cases. Prior to measurement, endothiapepsin was dialyzed in 100 mM acetate buffer. During the reference titration, the reference ligand solution consisting of 500 µM SAP114 3% DMSO and 100 mM acetate buffer with pH = 4.6 in the syringe, while the endothiapepsin solution was placed in the sample cell at a concentration of 50 µM with 3% DMSO. To perform the displacement titration, an identical solution of the reference ligand was loaded into the syringe, while the solutions of endothiapepsin (50 µM) with the different probe fragments (2 mM to 3 mM) were placed in the sample cell.

A	B	C	D	E	F	G	H	I	J	K	L	M	N
J.	Link to DOI	No.	ΔG1	ΔG2	ΔH1	ΔH2	- TΔS1	-TΔS2	ΔΔG	ΔΔH	-TΔΔS	Target	-xH2O
JMB	http://dx.doi.org/10.1016/j.jmb.2006.12.004	2->1	-35.4	-31.3	-25.6	-8.7	-9.8	-22.6	4.1	16.9	-12.8	AR	1
JMC	http://dx.doi.org/10.1021/jm300337q	3 -> 5	-54.2	-54.5	-36.2	-28.7	-18	-25.8	-0.1	3.75	-3.9	Thrombin	2
JMC	http://dx.doi.org/10.1021/jm300337q	8 -> 10	-47.1	-53.7	-13.8	-11.4	-33.3	-42.3	-3.3	1.2	-4.5	Thrombin	2
JMC	http://dx.doi.org/10.1016/j.jmb.2012.01.054	2 -> 1	-25.8	-31.7	-17.7	-13.6	-8.1	-18.1	-5.9	4.1	-10	Thrombin	1
JMB	http://dx.doi.org/10.1016/j.jmb.2009.04.051	5 -> 3a	-31.7	-34.8	-13.6	-28.5	-18.1	-6.3	-3.1	-15	11.8	Thrombin	1
JMC	http://dx.doi.org/10.1021/acs.jmedchem.5b00812	3 -> 2	-19.8	-19	-13.9	-29.7	-5.9	10.7	0.8	-16	16.6	Thrombin	1
JMC	http://dx.doi.org/10.1021/jm050424+	WT ->L300P Sorbinil	-37.9	-38.7	-51.8	-46.7	13.9	8	-0.8	5.1	-5.9	AR	1
JMB	http://dx.doi.org/10.1016/j.jmb.2009.06.016	4 -> 5	-40.1	-46.1	-38.7	-40.1	-1.4	-6.1	-6	-1.4	-4.7	Thrombin	1
CMC	http://dx.doi.org/10.1002/cmdc.201400013	4 -> 8	-37.9	-34.4	-21.4	-11.5	-16.5	-22.9	3.5	9.9	-6.4	Themolys	1
CMC	http://dx.doi.org/10.1002/cmdc.201400013	5 -> 4	-38.8	-37.9	-22.3	-21.4	-16.6	-16.5	0.9	0.9	0.1	Thermolys	1
JMB	http://dx.doi.org/10.1016/j.jmb.2010.02.007	3c -> 3e	-32.8	-37.4	-27	-22.7	-5.8	-14.7	-4.6	4.3	-8.9	Thrombin	1
JMB	http://dx.doi.org/10.1016/j.jmb.2010.02.007	4c -> 4e	-40.1	-42.9	-38.7	-34.5	-1.4	-8.4	-2.8	4.2	-7	Thrombin	1
BBA	http://dx.doi.org/10.1016/j.bbagen.2010.06.009	5 -> 3	-35.3	-37.6	-20.4	-31.1	-14.8	-6.5	-2.3	-11	8.3	Thermolys	1
JMB	http://dx.doi.org/10.1016/j.jmb.2012.01.054	8 -> 1b	-31.5	-35.4	-30.3	-37.1	-1.2	1.7	-3.9	-6.8	2.9	Thrombin	1
JMB	http://dx.doi.org/10.1016/j.jmb.2009.04.051												

Table S3: Excel-Table with the thermodynamic difference data of 14 pairs of closely related complexes where ligand binding is mainly determined by the pick-up or release of water molecules.

Column A: abbreviation for journal
Column B: DOI links to corresponding papers
Column C: Running No. of the ligands in the paper
Column D. E: $\Delta G1$ and $\Delta G2$ in kJ/mol
Column F. G: $\Delta H1$ and $\Delta H2$ in kJ/mol
Column H. I: -T$\Delta S1$ and -T$\Delta S2$ in kJ/mol
Column J: $\Delta\Delta G$ in kJ/mol
Column K: $\Delta\Delta H$ in kJ/mol
Column L: -T$\Delta\Delta S$ in kJ/mol
Column M: Target protein
Column N: Number of water molecules

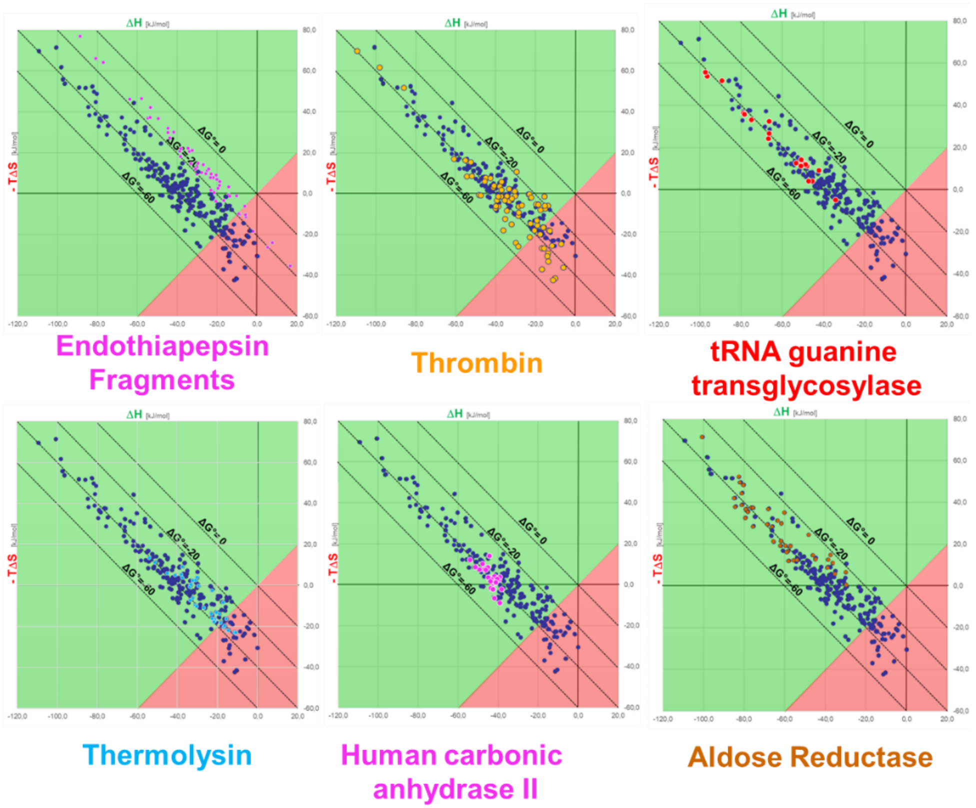

Figure S1: The ITC data of 266 protein-ligand complexes (dark blue) are plotted in a ΔH^0 versus $-T\Delta S^0$ diagram (cf. Figure 1). The data were collected using the different enzymes which are individually colored: Endothiapepsin (55 fragments added, pink, upper left), thrombin (orange, upper center), tRNA guanine transglycosylase (red, upper right), thermolysin (cyan, lower left), human carbonic anhydrase II (magenta, lower center), aldose reductase (brown, lower right). Ligand data were checked and corrected for superimposed buffer effects. Overall protonation state changes of protein residues of one enzyme uniformly observed for all ligands were not corrected, as they cancel out in a relative comparison of the binding profiles. However, if nitrogen-containing buffers were used (e. g. Hepes in case of thrombin) a pick-up of half a mole of protons by His 57 will result in an additional endothermic shift of the data by about 10 kJ/mol.

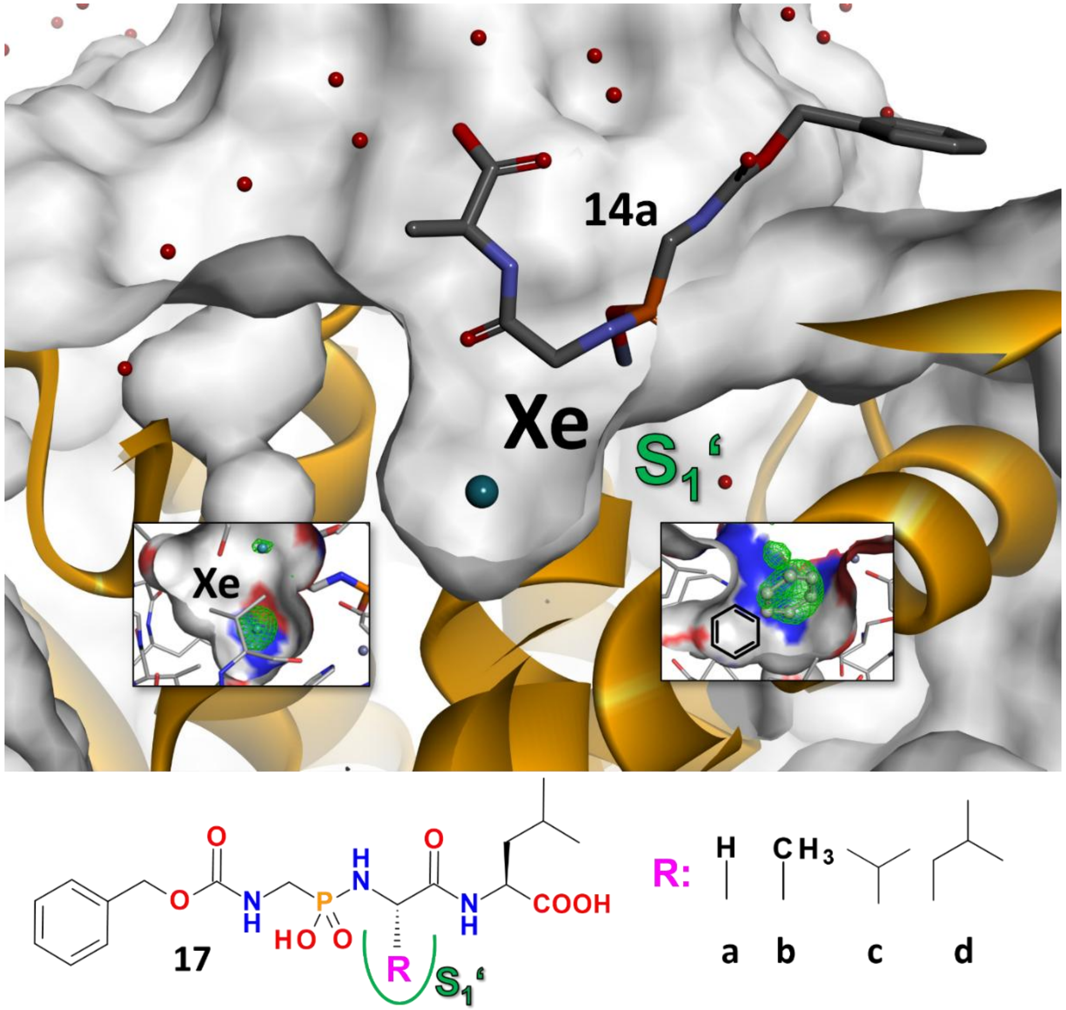

Figure S2: The S$_1$‘ pocket of thermolysin is virtually water-free. Hydrophobic side chains (a to d), which are attached to the ligand scaffold **17** increase binding by 41.000-fold primarily for enthalpic reasons (molecular representations as in Figure 2). The hydrophobic water-free pocket can accommodate as probes, a Xe atom or a benzene molecule (difference electron density is indicated by green meshes, and the polar regions of the S$_1$‘ pocket are highlighted by red and blue surface patches).
